# Self-Assembly of Lipid Mixtures in Solutions: Structures, Dynamics Processes and Mechanical Properties

**DOI:** 10.3390/membranes12080730

**Published:** 2022-07-23

**Authors:** Lingling Sun, Fan Pan, Shiben Li

**Affiliations:** Department of Physics, Wenzhou University, Wenzhou 325035, China; 20451025004@stu.wzu.edu.cn

**Keywords:** self-assembly, lipid mixture, phase behaviour, dynamics process, mechanical property

## Abstract

The self-assembly of lipid mixtures in aqueous solution was investigated by dissipative particle dynamics simulation. Two types of lipid molecules were modelled, where three mixed structures, i.e., the membrane, perforated membrane and vesicle, were determined in the self-assembly processes. Phase behaviour was investigated by using the phase diagrams based on the tail chain lengths for the two types of lipids. Several parameters, such as chain number and average radius of gyration, were employed to explore the structural formations of the membrane and perforated membrane in the dynamic processes. Interface tension was used to demonstrate the mechanical properties of the membrane and perforated membrane in the equilibrium state and dynamics processes. Results help us to understand the self-assembly mechanism of the biomolecule mixtures, which has a potential application for designing the lipid molecule-based bio-membranes in solutions.

## 1. Introduction

Lipid molecules contain one head chain and one or more tail chains, which can form various structures in solutions due to chain amphipathicity. The lipid architecture plays an important role in the formation of structures in self-assembly processes. The lipid chain with one hydrophilic functional group and one hydrophobic fatty acid group has a simple architecture; it possesses an inherent unique self-assembly structures in solutions and specific functions in biological processes, compared with phospholipid molecules that commonly have one head and two tail chains [1,2,3].

The lipids, with two or more tail chains, can self-assemble in solutions to produce a variety of symmetric and asymmetric structures. In addition to the most well-known structures, such as the layer, perforated layer, columnar and spherical vesicle structures, the amphiphilic peptides have been observed to self-assemble into new and not simple cubic structures [1,2,4,5,6]. These peptides can also form nanoscale folding or 3D periodic arrangements [6,7,8]. Considering the differences in the lipid structure caused by changing only one or two double bonds, scholars aim to optimise the physical or chemical properties of the structures [9]. The main energy storage molecule in organisms is triglyceride, which is composed of three acyl tail chains [10]. Kim et al. used a tuneable, phenomenological coarse-grained (CG) model to study triacylglycerol (TG) nucleation in a bilayer membrane [11]. Lipid molecules also function in forming complex chemical boundaries and dividing organelles, similar to biofilms in biological systems. Phospholipid membranes are usually composed of a head hydrophilic chain and two tail hydrophobic chains. Since phospholipids are the main components of biofilms, a large number of studies have focused on the exploration of phospholipid bilayer [12,13]. Hishida et al. used X-ray reflectivity and atomic force microscopy to investigate the morphology of dry phospholipid films and clarify the stacking structures of dry DOPC(1,2-dioleoyl-sn-glycero-3-phosphocholine) and DPPC(1,2-dipalmitoyl-sn-glycero-3-phosphocholine) films on a solid substrate [14]. Phospholipid vesicles have the behaviour similar to red blood cells in liquids; the effect of shear flow on phospholipid vesicles changes the phospholipid structure and thus could be used to study fluid dynamics in molecular dynamics (MD) predictions [15].

Other types of common lipids with a single tail and head chains have relatively simple chemical structures; these lipids include monoelaidin (ME), monovaccenin (MV), monolinolein (ML), monoamine (MA) and monoolein (MO), but their self-assembled structures are quite different from those in phospholipid molecules [16,17,18,19,20,21,22,23]. Also, the lipids with single tail and head chains can self-assemble into a series of bicontinuous cubic structures with liquid crystalline properties in the water environment [1]. The bicontinuous structures are similar to the recently reported continuous cubic phases (Ia3d, Pn3m and Im3m) in the block polymer system [24]. Cubic structures with Ia3d, Pn3m and Im3m symmetries were observed to be stable at a relatively higher temperature in the ME–water mixtures as determined by X-ray diffraction; phase diagrams were constructed based on water content and temperature [19]. The phase transition between the continuous structures and the other structures were reported in ML, ME and MO systems in water solutions by various experimental methods [18,22]. Lamellar structures were also reported in these lipids, such as ME and ML, in water solutions [18,19]; however, these lamellae with multiple layers differ from membrane structures with double layers. Mechanical properties were also reported in lipid membranes in experiments [25,26,27,28,29,30], and structural formations depend on the dynamics processes for lipid systems [31,32]. Hence, we need to understand the mechanical properties and dynamics processes for lipid membranes in aqueous solutions, especially for membranes constructed by lipids with one tail chain and one head chain.

The chain lengths of lipid molecules are exactly variant parameters in the experiments [33]; as such, the effects of chain lengths on the self-assembly of lipid molecules, especially when the two types of lipid molecules are mixed in solutions, should be explored. In this work, we use Dissipative Particle Dynamics (DPD) to investigate the self-assembly behaviour of lipid mixtures in aqueous solution, where the lipid is CG model consisting of a hydrophilic head chain and a hydrophobic tail chain. We introduce the model and method in Section 2. We then investigate the chain effects on the self-assembly structures, the corresponding dynamics processes and interfacial tensions for the self-assembled structures in Section 3. The summary is presented in Section 4.

## 2. Model and Methodology

### 2.1. CG Lipid Model

**Figure 1:**
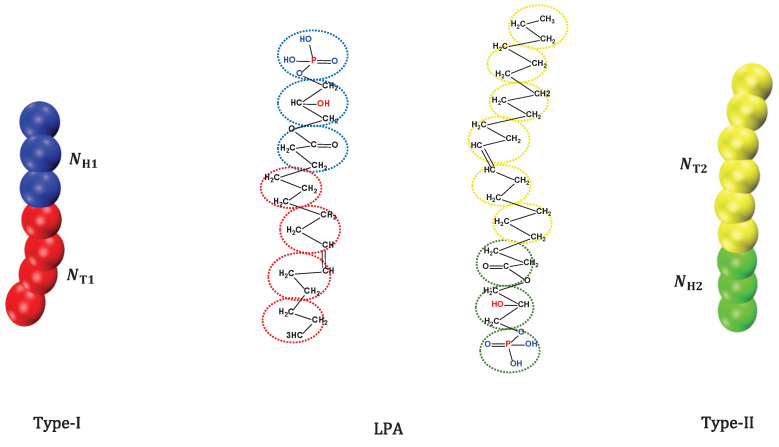
The schematic diagram for lipid molecule with one head group and one tail group. The leftmost side shows a schematic representation of the type-I lipid molecule, blue for the head beads and red for the tail beads. The rightmost side shows a schematic representation of the type-II lipid molecule, while green represents the head beads and yellow represents the tail beads. In the middle part is the illustration of lipid samples (single tail): lysophosphatidic acid (LPA).

The modelling of lipid molecules at all-atom level is very expensive in terms of computer memory resources due to the complexity of the lipid molecule. In this work, we used CG method, which treats a group of atoms as one bead, to model lipid molecules [34] (Figure 1). Here, we concentrated on lipid molecules with only one head chain and one tail chain in aqueous solution but belongs to two types of molecules, in contrast to other phospholipid molecules modelled with one head chain and two tail chains [4,35,36].

Figure 1 shows an example of the structure formed by two lipid molecule types (type-I on the left and type-II on the right). The hydrophobic tails (T) in type-I and type-II lipids are shown in red and yellow, and the hydrophilic head beads (H) in type-I and type-II lipids are shown in blue and green, respectively. Connectivity between the neighbouring beads is retained using the elastic harmonic force as follows [35,37]:

(1) $$F_{ij}=k_{s}\left(1-\frac{r_{ij}}{r_{s}}\right){\hat{r}}_{ij},$$

where $k_{s}$ is the spring constant between the two successive *i*-th and *j*-th beads, $r_{s}$ and $r_{ij}$ are the usual equilibrium bond length and the distance between the two beads, respectively. Here, ${\hat{r}}_{ij}$ is the unit vector, where ${\hat{r}}_{ij}$=$r_{ij}/\left|r_{ij}\right|$, with $r_{ij}$=$r_{i}-r_{j}$ and $r_{\mathrm{ij}}$=$\left|r_{ij}\right|$. Here, we set $k_{s}=120.0$ and $r_{s}=0.7r_{c}$ in reference to the study of Groot et al. [38]. In addition, an angle exists between every three beads, and bending force is given by:

(2) $$F^{\theta }=-\nabla \left[k_{\theta }{\left(\theta -\theta_{0}\right)}^{2}\right].$$

Resistance to bending forces exists between two adjacent bonds, where $k_{\theta }$ is the bending constant, and θ is the inclination angle with an equilibrium state $\theta_{0}$. In the present work, we selected parameters similar to those in the previous studies [39]. We used $k_{\theta }=6.0$ and $\theta_{0}=\pi$ for three consecutive beads in each chain of lipid molecule to represent a linear chain; it is for three consecutive head or tail beads, or it may be the bending force of the connection between the head and tail beads.

### 2.2. Dissipative Particle Dynamics

DPD is based on the CG model and obeys Newton’s law. DPD was originally developed to simulate the hydrodynamic behaviour in mesoscopic complex fluids [40,41,42] and was refined by Basan [43]. In the present work, we only briefly describe DPD simulation. According to Newton’s second law of mechanics, it holds $\frac{dv_{i}}{dt}=f_{i}$ and $\frac{da_{i}}{dt}=v_{i}$, where *t* is time. For example, between a pair of *i*-th and *j*-th beads, the total forces on the *i*-th bead can be calculated as follows [44]:

(3) $$m_{i}\frac{d^{2}r_{i}}{dt^{2}}=F_{i}=\sum_{i\neq j}\left(F_{ij}^{C}+F_{ij}^{D}+F_{ij}^{R}\right).$$

Each DPD bead interacts with other beads within a certain cut-off distance $r_{c}$, i.e., $r_{ij}r_{c}$. The forces acting on each bead are composed of three components, namely, conservation force, dissipative force and random force. The conservation force, $F_{ij}^{C}=a_{ij}w\left(r_{ij}\right){\hat{r}}_{ij}$ is derived from a potential, where $a_{ij}$ is the maximum repulsion between the *i*-th and *j*-th beads. Here, $w\left(r_{ij}\right)$ is a dimensionless weight function, which can be written as:

(4) $$w\left(r_{ij}\right)=\left\{\begin{array}{cc} 1-\frac{r_{ij}}{r_{c}} & r_{ij}r_{c}\\ 0 & r_{ij}r_{c} \end{array}\right..$$

In Equation $\left(3\right)$, the dissipative force, $F_{ij}^{D}=-\gamma w^{2}\left(r_{ij}\right)\left({\hat{r}}_{ij}\cdot v_{ij}\right){\hat{r}}_{ij}$, represents viscous forces between DPD beads, where the parameter γ is a viscosity coefficient. This force is affected by relative position $r_{ij}$ and relative velocity $v_{ij}$, with $v_{ij}$ = $v_{i}-v_{j}$. The last term is random force, $F_{ij}^{R}=\sigma w\left(r_{ij}\right)\zeta_{ij}\Delta t^{-0.5}{\hat{r}}_{ij}$, representing the effects of thermal fluctuations. Here, σ is the noise amplitude, while $\zeta_{ij}$ is a random variable with Gaussian distribution and unit variance. Dissipative and random forces cannot be chosen independently; at a constant temperature, σ and γ are related by:

(5) $$\sigma^{2}=2\gamma k_{B}T,$$

where $k_{B}$ is the Boltzmann constant, *T* is the equilibrium temperature and $k_{B}T$ is the energy unit. In the present work, we use σ=3.0 and γ=4.5, which preserve the momentum and achieve a thermostatic effect [35].

### 2.3. Simulation Parameters

Several parameters were used in the simulation systems. Firstly, Groot and Warren’s velocity–Verlet algorithm [45] was used in DPD simulation. The formula is as follows:

(6) $$\begin{array}{c} r_{i}\left(t+\Delta t\right)=r_{i}\left(t\right)+\Delta tv_{i}\left(t\right)+\frac{1}{2}{\left(\Delta t\right)}^{2}f_{i}\left(t\right)\\ {\tilde{v}}_{i}\left(t+\Delta t\right)=v_{i}\left(t\right)+\lambda \Delta tf_{i}\left(t\right)\\ f_{i}\left(t+\Delta t\right)=f_{i}\left(r\left(t+\Delta t\right),{\tilde{v}}_{i}\left(t+\Delta t\right)\right)\\ v_{i}\left(t+\Delta t\right)=v_{i}\left(t\right)+\frac{1}{2}\Delta t\left(f_{i}\left(t\right)+f_{i}\left(t+\Delta t\right)\right). \end{array}$$

Here, the time step is selected to be Δt=0.01τ, similar to the previous studies [46]. We can obtain that $\tau =1.88ns=\sqrt{mr_{c}^{2}/k_{B}T}$, the natural unit of time, where the diffusion constants in the plane are considered from the experiments [47,48]. In brief, *m* is the mass of one bead and scaled to be the unit mass, and $r_{c}$ is the cut-off distance with $r_{c}$ = ${\left(\rho V_{b}\right)}^{1/3}$, which is scaled to be the unit length. Here, we assume that the volume $V_{b}$ of one DPD bead and the density ρ of the entire system are 0.03 nm${}^{3}$ and 3, respectively [49]. The $r_{c}$ of the bead in the lipid–water mixture is about 0.5 nm.

**Figure 2:**
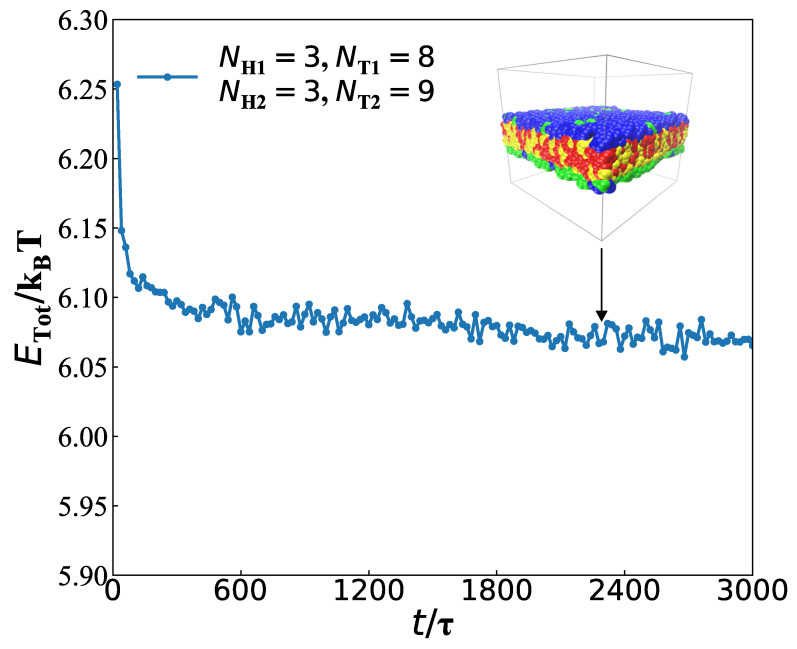
An example of obtaining equilibrium state in a dynamics process with parameter $N_{H}1=3$, $N_{T}1=8$, $N_{H}2=3$ and $N_{T}2=9$, the total energy $E_{\mathrm{Tot}}/k_{B}T$ as a function of time is shown.

In the current DPD model, considering the periodic boundary condition [35], the volume of the simulation cube box is $V=L_{x}\times L_{y}\times L_{z}=30r_{c}\times 30r_{c}\times 30r_{c}$. Since the finite size effect is not negligible [50], we adjust the box size from 25$r_{c}$ to 35$r_{c}$ to avoid the finite size effect. The optimised box size is $L=30r_{c}$, which is similar to those of our simulations [35]. In general, the whole system was carried out under the NVT ensemble system by using the parallel simulator LAMMPS (large-scale Atomic/Molecular Massively Parallel Simulator) [51]. As shown in Figure 2, when the running time step is 200,000, an equilibrium structure can be obtained [52]. About 300,000 DPD steps were performed in all simulations to ensure the acquirement of equilibrium structures. Based on Flory–Huggins theory [53,54], the repulsive interaction parameters can be expressed as [55]:

(7) $$\chi =0.286\left(a_{ij}-a_{ii}\right).$$

We took the repulsive interaction parameters $a_{ii}$ = 25 for the same type of beads and $a_{ij}$ = 100 for different types of beads. Hence, the Flory coefficient between two different beads is about 21.45, which is located in the strong segregated regime. For a description in detail, we list the interaction parameters in a table, as shown in Table 1, in which the other parameters were also list. Thus, the strong repulsive interactions between the tail bead and water bead probably cause the lipid molecules to become insoluble in water. Indeed, many lipids with one head chain and one tail chain are not water-soluble in the experiments, which should depend on the experimental methods to process the self-assembly [20,21,22,23].

To observe the effect of chain length, we fixed the other parameters in the current simulations. The total number of beads in our simulation system is fixed at N= 81,000, and the chain numbers are the same as $n_{1}=n_{2}=900$ for the type-I and type-II lipids. The head beads are set to be $N_{H1}=N_{H2}=3$ in the individual type-I and type-II lipid chains. Our purpose is to observe the phase behaviour by changing the numbers of tail beads $N_{T1}$ and $N_{T2}$ for type-I and type-II lipid chains, respectively, under a constant chain number concentration *n*, where $n=(n_{1}+n_{2})/V$. Then, the total bead number can be expressed as $N=n_{1}\left(N_{H1}+N_{T1}\right)+n_{2}\left(N_{H2}+N_{T2}\right)+N_{W}$. Here, the number of water beads $N_{W}$ is a variable when $N_{T1}$ and $N_{T2}$ change, in order to keep the constant *N*.

Since the dynamics process depends on the processing pathway [56], the initial state can affect the dynamics process during the self-assembly of lipid molecules. Usually, we input several different initial structures, such as the lamellar and spherical structures, as well as the random inputting, for a given system parameter set in the simulations. Then, we choose the output structure with the minimum energy as the stable state for the given system parameters in the simulations, which was similar to our previous simulations [39]. For example, we input the initial lamella-like structure to search for the final stable membrane by trying many times in the current simulations. Specifically, our initial biayer-like inputs were prepared in the simulation boxes, where the type-I lipids are on the upper layer and type-II lipids on the lower layer, in order to observe the mixing mechanism in the dynamic processes. Considering the varieties of simulation samples, we prepared different initial layer-like inputs in order to achieve the best final structure. Here, we note that the initial states are not mixed, which are similar to the initial arrangements in the previous simulations and experiments [57,58,59,60]. In these works, the various initial structures can be obtained by the various experimental methods. The example is that the substrate-supported lipid bilayer assemblies can be prepared on atomically flat mica receptions by the standard vesicle fusion technique [58].

## 3. Results and Discussion

**Figure 3:**
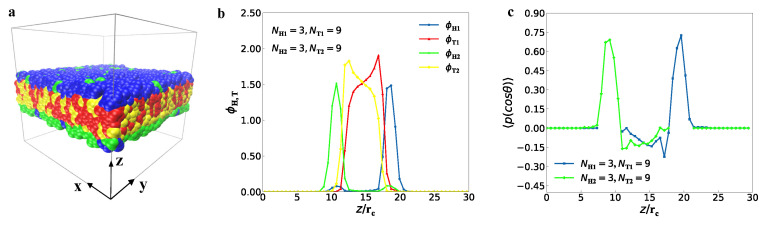
Representative membrane structure (**a**) in solutions with type-I lipid chain parameters of $N_{H}1=3$, $N_{T}1=9$ and the type-II lipid chain parameters of $N_{H}2=3$, $N_{T}2=9$. The corresponding density profiles of (**b**) is shown in the middle and (**c**) the rightmost side shows a distribution of order parameters.

**Figure 4:**
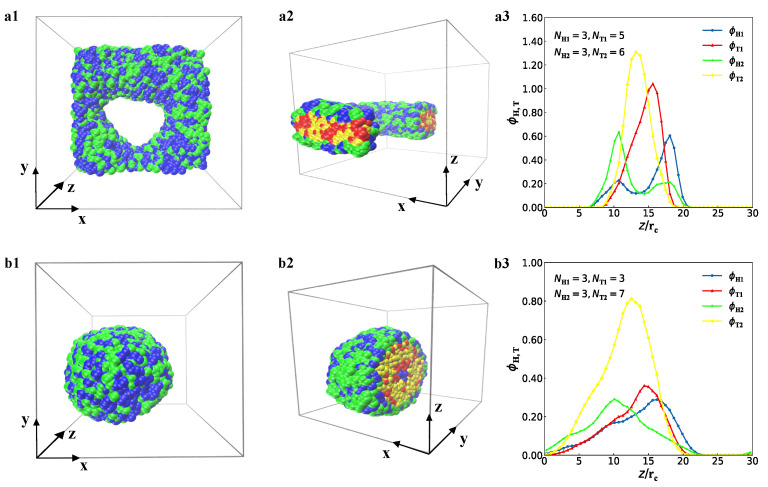
Representative perforated membrane and spherical vesicle in solutions. Sectional views and corresponding density profiles are shown. (**a1**) The side view of the perforated membrane with parameters of $N_{H}1=3$, $N_{T}1=5$, $N_{H}2=3$, and $N_{T}2=6$. (**a2**) A cross-sectional view of the perforated membrane. (**a3**) The density distribution of the perforated membrane along the *z*-direction. (**b1**) The side view of the vesicle with parameters of $N_{H}1=3$, $N_{T}1=3$, $N_{H}2=3$, and $N_{T}2=7$. (**b2**) A cross-sectional view of the vesicle. (**b3**) The density distribution of the vesicle along the *z*-direction.

**Figure 5:**
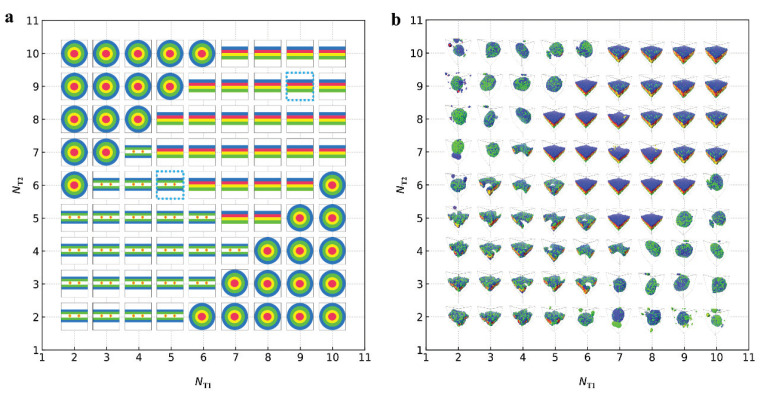
The phase diagrams of the structures self-assembled from the lipid molecules in solutions. (**a**) Phase diagram arranged with the phase symbols. The phase symbols 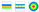 represent the bilayer membranes, perforated bilayer membranes and spherical vesicles, respectively. (**b**) Phase diagram arranged with the real snapshots. All the phases are arranged by the numbers of tail beads for two types of lipid chains.

**Figure 6:**
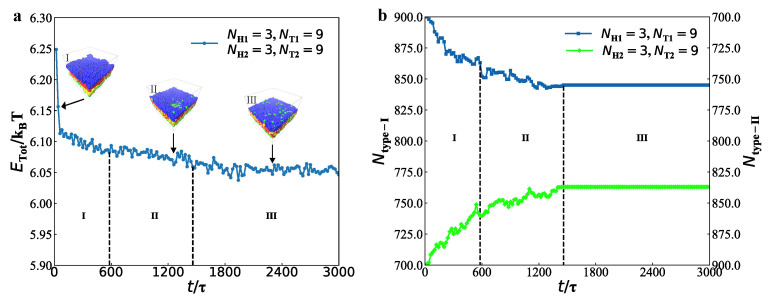
Energy and chain numbers as functions of the time in the dynamics processes for the membrane with parameters of $N_{H}1=3$, $N_{T}1=9$, $N_{H}2=3$ and $N_{T}2=9$. (**a**) Energy as function of time. (**b**) Chain numbers as functions of time.

**Figure 7:**
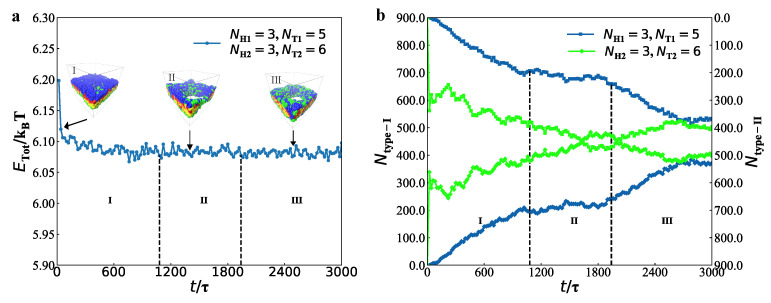
The analysis about dynamics processes for the perforated membrane with parameters of $N_{H}1=3$, $N_{T}1=5$, $N_{H}2=3$ and $N_{T}2=6$. (**a**) Energy as function of time. (**b**) Chain numbers as functions of time.

**Figure 8:**
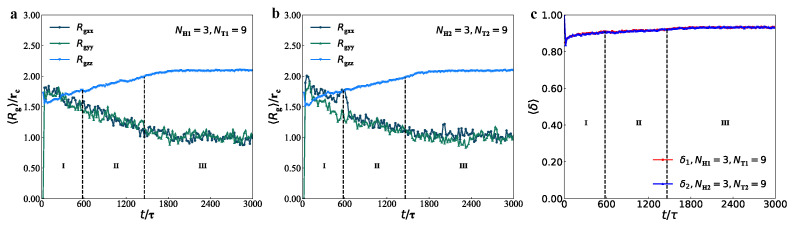
The average radius of gyration $\langle R_{g}\rangle$ of (**a**) type-I lipid chains and (**b**) type-II lipid chains in the bilayer membranes. (**c**) The shape factors of type-I lipid chains $\delta_{1}$ and type-II lipid chains $\delta_{2}$ in the bilayer membranes.

**Figure 9:**
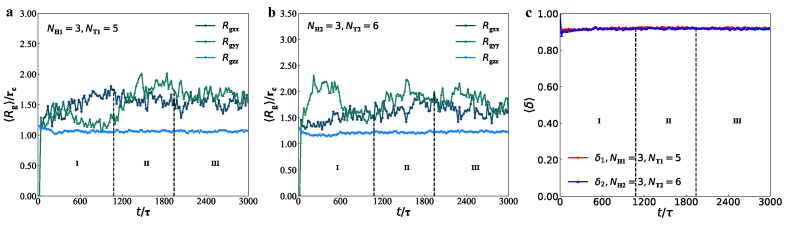
The average radius of gyration $\langle R_{g}\rangle$ of (**a**) type-I lipid chains and (**b**) type-II lipid chains in the perforated membranes. (**c**) The shape factors of type-I lipid chains $\delta_{1}$ and type-II lipid chains $\delta_{2}$ in the perforated membranes.

**Figure 10:**
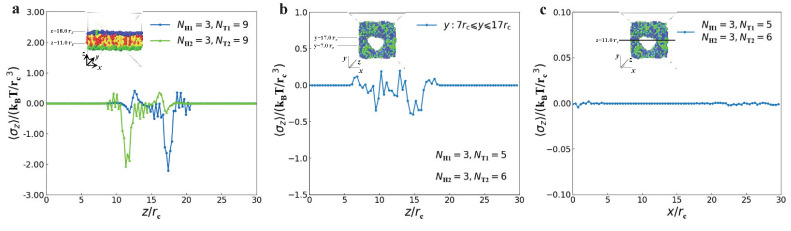
The interfacial tension $\langle \sigma_{z}\rangle$ as functions of positions along different directions for the membrane and perforated membrane, respectively. (**a**,**b**) The interfacial tensions are along the *z*-direction, and (**c**) goes along the *x*-direction.

**Figure 11:**
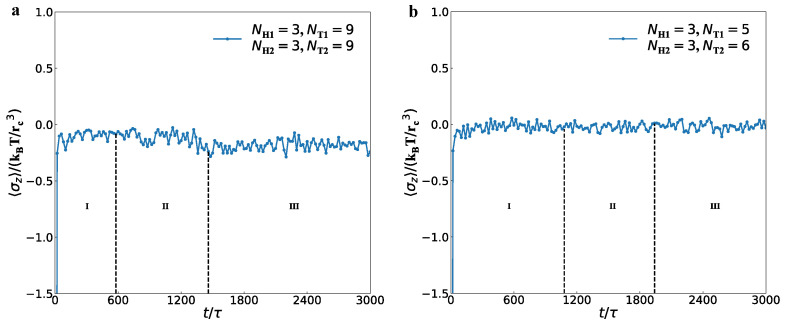
The interfacial tension $\langle \sigma_{z}\rangle$ varies with the time step for (**a**) the membrane and (**b**) perforated membrane, respectively.

In this section, we analysed and discussed the results from the DPD simulations. The self-assembled structures of lipid mixtures were described (Figure 3 and Figure 4) and the phase diagrams (Figure 5) were plotted in Section 3.1. We analysed the dynamics processes under the certain parameters (Figure 6, Figure 7, Figure 8 and Figure 9) and discussed the physical mechanism concerning the formation of such structures in Section 3.2. We considered the interfacial tensions for the membranes and perforated membranes in the equilibrium states and dynamics processes (Figure 10 and Figure 11) in Section 3.3.

### 3.1. Typical Structures

We observed three typical structures in the self-assembly of lipid mixtures in the aqueous solution, i.e., the bilayer membrane, perforated bilayer membranes and spherical vesicles. First, we observed the bilayer membrane (Figure 3), where the parameters are $N_{H}1=3$, $N_{T}1=9$, $N_{H}2=3$ and $N_{T}2=9$, similar to those in the previous works [61,62]. At first glance, the upper layer in the bilayer membrane is made up of type-I lipid molecules, while lower layer consists of type-II lipids (Figure 3a). To observe the structure clearly, here we did not plot the water molecules. In the side view, the tail chains of type-I and type-II lipids are concentrated within the inner region of the bilayer membrane due to the hydrophobicity of tail chains. As the tail chains are long enough, they overlap in the inner region, as shown in Figure 3b, where the bead density distributions are plotted for the tail and head chains of type-I and type-II lipids, respectively. The type-I and type-II head chains have two separate peaks in the bead density distributions, indicating that the double layers are formed in the membrane. The thickness of this membrane is about 11$r_{c}$, and the center of membrane is located at *z* = 14.5$r_{c}$. The detailed data analysis showed that the chain numbers of type-I and type-II lipids are 845 and 63 in the upper layer, respectively, and the total chain number is 908. In the lower layer, the chain numbers of type-I and type-II lipids are 55 and 837, respectively, with a total chain number of 892. These data clearly show the membrane is a mixed structure with asymmetric distributions for type-I and type-II lipids in the upper and lower layers.

To illustrate structures in more detail, we introduce the order parameter as follows [63]:

(8) $$\langle P\left(\cos\theta \right)\rangle =\langle \frac{3}{2}\cos^{2}\theta -\frac{1}{2}\rangle .$$

The order parameter is usually used in the eigen anisotropic materials, such as liquid crystal materials [62]. Here, θ represents the included angle between the chain and the *z*-axis direction, and the angle brackets represent the mean value over the ensemble. In particular, when the order parameter is equal to 1, the direction of the chain parallels to the *z*-axis; when the order parameter is greater than 0 and less than 1, the chain is partially aligned with the *z*-axis; when the order parameter is equal to 0, the chain is distributed randomly; when the order parameter is equal to −0.5, the chain direction is perpendicular to the *z*-axis [64]. According to Equation (8), we plotted the order parameters for the head beads of two types of lipid chains (Figure 3c). The order parameters have the maximum value of about 0.75 for type-I and type-II head chains, showing that the head chains are in good orders, nearly parallel to the z-direction, the normal direction of membrane. The results also showed that the lipid chains have weak orders in the center zones for the membranes.

Figure 4 shows the perforated bilayer membrane and spherical vesicles. For the perforated bilayer membrane, the top view, side view and bead density profiles are shown in Figure 4a(1–3), respectively, where the parameters are $N_{H}1=3$, $N_{T}1=5$, $N_{H}2=3$ and $N_{T}2=6$. The data analysis showed that the thickness of this perforated membrane is about 10$r_{c}$. The head chains are distributed in the membrane surfaces, while the tail chains are located inside the membrane due to the hydrophilicity of the head beads and the hydrophobicity of the tail beads (Figure 4a(1)). In the side view (Figure 4a(2)), the head chains of type-I and type-II lipids are intersected and mixed with each other on the membrane surface, but the two types of tail chains overlap inside the membranes. The double layers in the perforated membranes are not perfect layer. The perforated layer was also reported in the previous experiments or simulations, which has potential applications in drug delivery during biological processes [65,66,67]. We plotted the bead density profiles along the *z*-directions for the perforated biayer membrane, as shown in Figure 4a(3), where several peaks appear in the four types of beads. The type-I lipid has longer tail chain than that of type-II lipid, which leads to a larger peak. This indicates that the chains of type-I lipid are almost concentrated inside the membranes. The double peaks for the head chains illustrated the two types of head chains on the membrane surface. The data analysis showed that the chain numbers of type-I and type-II lipids are 529 and 397 in the upper layer, respectively, while are 371 and 503 in the lower layer, clearly indicating that the perforated membrane is a mixed structure. The perforated bilayer membranes have been reported in the previous simulations [68,69], where the surfaces were constructed by one type of molecules. Here, we observed that the perforated membranes are mixed with the two types of lipid molecules on the membrane surfaces.

The spherical vesicle is another mixed structure in the current simulations, as shown in Figure 4b, where the parameters are $N_{H}1=3$, $N_{T}1=3$, $N_{H}2=3$, and $N_{T}2=7$. The data showed that the diameter of the vesicle is about 22$r_{c}$. From the top view in Figure 4b(1), the head chains have a trend to be distributed on the outer surface for the vesicle. On this surface, two types of lipid head chains intersect each other due to the adsorption interactions between the solution molecules and the lipid head chains. To observe the inner structures, we plotted the cross-sectional view for the spherical vesicle (Figure 4b(2)). The inner structure of spherical vesicles can be clearly observed, where the inner core is also composed of two types of tail beads, which are hydrophobic beads, together to form a spherical closed bilayer structure. Furthermore, we also plotted the bead density profiles along the radial direction (Figure 4b(3)). The four types of beads are all in a relatively concentrated arrangement, where the maximum appears in different positions. The type-II lipid chain has more tail beads and the highest density, which is severely squeezed and arranged, while the two peaks for type-I and type-II head chains indicate that these chains are mixed on the surfaces of the spherical vesicle. The evidence is that the chain numbers of type-I and type-II lipids are 895 and 644 in the outer layer, respectively. Due to the short length of type-I lipid chain and the longer length of type-II lipid chain, this original asymmetry has a slightly irregular shape in the self-assembly, and the aggregation behaviour becomes more obvious in the aqueous solution. The lipid density can be also captured by the SuAVE software, which is a tool for analysing the curvature-dependent properties [70]. Here, we used lipid densities along radial directions to analyse the vesicle structure, where the vesicle formation in each case is similar to a cell reported in a previous work [71].

Here, we investigated the phase behaviour by varying the tail chain lengths. We constructed the phase diagram (Figure 5), where the numbers of tail beads, $N_{T}1$ and $N_{T}2$, vary from 2 to 10, and the membrane, perforated membrane and vesicles are arranged with $N_{T}1$ and $N_{T}2$. First, we constructed the phase diagram with simple phase symbols, as shown in Figure 5a. As a whole, the phase space can be divided into three sub-spaces, where the membranes, perforated membranes and vesicles are located by one another. The phase diagram possesses the symmetry about the diagonal line with $N_{T}1=N_{T}2$, as shown in Figure 5a. This finding is logical because the role of tail chain length can be completely exchanged for type-I and type-II lipids. Such symmetries were observed in the diblock copolymer system, where the role of two blocks can be exchanged either under confinement or in bulk [72,73,74]. The spherical vesicles are located in the corner, where the tail chain lengths are relatively asymmetric. As such, vesicles are easily formed for the lipid molecules in the solution when the chain lengths have obvious asymmetry, consistent with our previous works [39,75] and the experimental results [11,76]. The membranes are located near the diagonal line and in the upper corner of phase diagram, in which the tail chain lengths are relatively large and comparative for type-I and type-II lipid chains. The perforated membranes are also distributed near the diagonal line in the phase diagram, but the tail chain length is relatively short for type-I and type-II lipid chains. Membranes are usually formed in solutions when the chain lengths are symmetric for two blocks in diblock copolymers [4]. Here, we observed the unusual distributions for the perforated membranes in the phase diagrams, where the tail chains are shorter than the head chains probably due to the intersections between the two types of head chains on the membrane surfaces. This intersection breaks the entropy–enthalpy balances between the systems, which causes such distribution in the phase diagram.

Then, we plotted the phase diagram with real snapshots, as shown in Figure 5b. Certainly, the phase boundaris are the same as those in the phase diagram with phase symbols. However, the phase diagram with real snapshots provides more information about the phase behaviour for the self-assembly of lipid molecules. For example, one can observe the mixtures of type-I and type-II lipid chains in the upper layers from these real snapshots in the phase diagram. Under a special condition, for example, when $N_{H1}=N_{H2}=3$ and $N_{T1}=N_{T2}=2$, we need $N_{W}=$ 72,000 water beads in the simulation box. Under this condition, the numbers of type-I and type-II lipid beads are the same as 4500, which are not large enough to participate in the self-assembly, leading to an unperfected perforated membrane, as shown in the real snapshots in Figure 5b. The detailed observations on the real snapshots indicated that the membrane thickness and vesicle diameter increase as $N_{T1}$ and $N_{T2}$ increase. This is reasonable because there are more lipid beads participate into the self-assembly as $N_{T1}$ and $N_{T2}$ increase. Thus, the phase diagram with the real snapshots provides more information about the phases, while the phase diagram with phase symbols provides a guidance for the approximate phase boundaries.

### 3.2. Dynamics Processes

DPD not only enables us to investigate the equilibrium states but also allows us to analyse the dynamics processes. In this subsection, we concentrated on the structural formation processes for the membranes and perforated membranes in the aqueous solutions by analysing several parameters such as system energy, mean-square radius of gyration and shape factor. We took the membrane with parameter $N_{H}1=3$, $N_{T}1=9$, $N_{H}2=3$ and $N_{T}2=9$, and the perforated membrane with parameter $N_{H}1=3$, $N_{T}1=5$, $N_{H}2=3$ and $N_{T}2=6$, as two examples to evaluate the dynamics processes.

First, we discussed the dynamics processes for the membranes with parameters of $N_{H}1=3$, $N_{T}1=9$, $N_{H}2=3$ and $N_{T}2=9$ by analysis of energy and chain number (Figure 6).

As shown in Figure 6a, we analysed the energy evolution in the dynamics process, in which the typical structures were also inserted. The structural evolution process can be divided into three stages, i.e., the initial stage, the adjustment stage and the stable stage. When t=0τ, the energy is $E_{\mathrm{Tot}}/k_{B}T=6.25$, and the average energy $\langle E_{\mathrm{Tot}}/k_{B}T\rangle =6.12$ in the initial stage. The energy decreases rapidly and has a violent oscillation in the initial stage. The initial stage is short due to the special initial chain conformation. This is not a coincidence but a common phenomenon similar to the previous experiment and simulation [75]. The different initial inputs, i.e., the random and layer conformation inputs, differ in the time of forming stable state. Namely, the time they reach the equilibrium is different, but both finally reach the target structure [39]. At the second stage with t=580τ−1460τ, the average energy slightly decreases to be $\langle E_{\mathrm{Tot}}/k_{B}T\rangle =6.08$. The weak decrease in energy still shows a fluctuation, indicating that the chain is constantly adjusting and evolving. Finally, for a long period of t=1460τ−3000τ, the system evolves into a stable bilayer, with the energy of $\langle E_{\mathrm{Tot}}/k_{B}T\rangle =6.05$. At this stage, the state of the bilayer membrane is almost unchanged. Here, there is a competition between the entropies from the chain conformations and the interaction energies among the chains and water. Such a competition determines the final stable state in the dynamics processes [77,78,79,80].

This competition obviously exhibits in the chain conformations, which leads to the mixtures between the type-I and type-II lipid chains. In order to observe the mixing process, we plotted the chain numbers of type-I lipids in the upper layer, $N_{\mathrm{type}-I}$, and type-II lipids in the lower layer, $N_{\mathrm{type}-\mathrm{II}}$, in the dynamics processes, as shown in Figure 6b. In the beginning, the initial layer-like structure is completely unmixed, where all of type-I lipids distributes in the upper layer and type-II lipids in the lower layer. Then, a small amount of lipids begin to mix with each other, where both the $N_{\mathrm{type}-I}$ and $N_{\mathrm{type}-\mathrm{II}}$ decrease in the upper and lower layers, respectively. In the initial stage, there are average 875 chains of type-I lipids in the upper layer, while the average chain number of type-II lipids is also 875 in the lower layer. The mixture in this stage shows a symmetric feature, clearly shown in Figure 6b. In the second stage, $N_{\mathrm{type}-I}$ and $N_{\mathrm{type}-\mathrm{II}}$ decrease smoothly due to the adjustment. In this adjustment stage, the average $N_{\mathrm{type}-I}$ and $N_{\mathrm{type}-\mathrm{II}}$ are 849 and 847 in the upper and lower layers, respectively. When the system develops into the stable stage, the chain numbers of type-I and type-II lipids maintain unchanged values in the upper and lower layers, respectively, i.e., $N_{\mathrm{type}-I}$ = 845 and $N_{\mathrm{type}-\mathrm{II}}$ = 837. Here, the chain number enables us to effectively analyse the mixture processes in the dynamics processes.

Then, we discussed the dynamics processes of perforated membrane (Figure 7), with the parameter of $N_{H}1=3$, $N_{T}1=5$, $N_{H}2=3$ and $N_{T}2=6$. Similarly, the evolution process can be divided into three parts, the initial stage, the adjustment stage and stable stage.

Specifically, the energy is $E_{\mathrm{Tot}}/k_{B}T=6.20$ at t=0τ, lower than that in the membrane structure. Similar to the membrane case, the initial input conformation is the unmixed layer input. This stage continues about 1080τ, and the average energy is to be $\langle E_{\mathrm{Tot}}/k_{B}T\rangle =6.09$ with a rapid decreasing. A tiny notched-hole with a diameter of about 16.2$r_{c}$ appears in the right region of the perforated membrane at t=1080τ, as shown in Figure 7a. Then, the system develops into the adjustment perforation stage, maintaining about 860τ, with the average energy of $\langle E_{\mathrm{Tot}}/k_{B}T\rangle =6.08$. The inserted conformation showed that the two types of lipids are not clearly aligned, but mixed to a certain extent. Meanwhile, the hole appears in the middle region and assumes a more formalized shape at t=1940τ, with a diameter of about 17.3$r_{c}$. Obviously, the third stage maintain with t=1940τ−3000τ, and the average energy is $\langle E_{\mathrm{Tot}}/k_{B}T\rangle =6.07$, which is close to the final energy output in membranes. In this stage, its diameter and position maintain unchanged, with the diameter fixed at 17.0$r_{c}$. Here, the dynamics processes for the perforated membrane are similar to those observed about the perforated bilayer membranes in the biological systems [81].

We plotted the chain numbers of type-I and type-II lipids in the upper and lower layers, respectively, as shown in Figure 7b. Here, the curves in dark blue denote the numbers of type-I lipids in the upper and lower layers, repectively, corresponding to the left vertical coordinate, while the curves in bright green represent the numbers of type-II lipids in the upper and lower layers, corresponding to the right vertical coordinate. The $N_{\mathrm{type}-I}$ and $N_{\mathrm{type}-\mathrm{II}}$ clearly demonstrate the mixing processes for the perforated membrane in the initial, adjustment and stable stages. Specifically, there were average 787 type-I and 325 type-II lipid chains in the upper layer, and 113 type-I and 575 type-II lipid chains in the lower layer in the initial stage. For the adjustment stage, the average $N_{\mathrm{type}-I}$ and $N_{\mathrm{type}-\mathrm{II}}$ are 685 and 434 in the upper layer, respectively. While average $N_{\mathrm{type}-I}$ and $N_{\mathrm{type}-\mathrm{II}}$ are 571 and 407 in the upper layer, respectively, in the stable stage. It can be easily concluded that during three dynamics processes, the numbers of different types of lipids on both sides have changed significantly. In recent studies, there were medical research theories for lipid membranes with gaps [82,83], and the biofilm partial phase separation according to the basic principle of lipid rafts hypothesis [84,85]. Here, we observed the dynamics processes for the membrane and perforated membrane to analyse how the pore formation and the mixtures of two types of lipid chains in the dynamics processes.

Then, we turned to investigate the dynamics processes by mean-square radius of gyration. The mean-square radius of gyration is an important parameter to describe the polymer size, which can be expressed as [86]:

(9) $$\langle R_{g}^{2}\rangle =\frac{1}{N}\sum_{i=1}^{N}\langle {\left(r_{i}-r_{c}\right)}^{2}\rangle ,$$

where $r_{c}=N\sum_{j=1}^{N}r_{j}$, represents the coordinates of the chain centre of mass. In order to show its characteristics more accurately, we introduce the radius of gyration tensor $R_{g}^{2}$:

(10) $$R_{g}^{2}=\left(\begin{array}{ccc} R_{gxx}^{2} & R_{gxy}^{2} & R_{gxz}^{2}\\ R_{gyx}^{2} & R_{gyy}^{2} & R_{gyz}^{2}\\ R_{gzx}^{2} & R_{gzy}^{2} & R_{gzz}^{2} \end{array}\right).$$

Here, the elements $\langle R_{g\alpha \beta }^{2}\rangle =\frac{1}{N}\sum_{i}\langle \left(r_{i,\alpha }-r_{c,\alpha }\right)\left(r_{i,\beta }-r_{c,\beta }\right)\rangle$,α,β∈{x,y,z}, and *N* in equation represent the number of chains, and $r_{i,x}$ in brackets represents the *x* coordinate of the *i*-th bead.

We took the same example membrane to describe the dynamics processes by the radius of gyrations, as shown in Figure 8, where the parameters are set to be $N_{H}1=3$, $N_{T}1=9$, $N_{H}2=3$ and $N_{T}2=9$.

We plotted three components of mean-square radius of gyrations $\langle R_{gxx}\rangle$, $\langle R_{gyy}\rangle$ and $\langle R_{gzz}\rangle$ for the type-I and type-II lipids, as shown in Figure 8a,b, respectively. For the type-I and type-II lipids, the $\langle R_{gxx}\rangle$, $\langle R_{gyy}\rangle$ and $\langle R_{gzz}\rangle$ have the similar behaviour in the dynamics processes, where $\langle R_{gzz}\rangle$ increase while $\langle R_{gxx}\rangle$ and $\langle R_{gyy}\rangle$ decrease in the dynamics processes; the three components tend to have stable values. Such evolutions suggest that type-I and type-II lipid molecules are relaxed along the membrane planes but become more orderings in the normal directions of membranes. Specifically, the average values of $\langle R_{gxx}\rangle$ and $\langle R_{gyy}\rangle$ are relatively close and smaller than those of $\langle R_{gzz}\rangle$, and the oscillation amplitude of lipid molecules in the *x*- and *y*-directions is also larger than that in the *z*-direction. Hence, the lipid molecules are relatively dense in the *x*- and *y*-directions and relatively stretched in the *z*-direction. This finding is consistent with their free energy and the corresponding structural variations, similar to the formation process of self-assembly of lipid molecules in the previous work [87].

We introduced the shape factor to describe the chain shapes, according to the radius of gyration tensor, which can be expressed as [88,89,90]:

(11) $$\langle \delta \rangle =1-3\langle \frac{L_{1}^{2}L_{2}^{2}+L_{2}^{2}L_{3}^{2}+L_{1}^{2}L_{3}^{2}}{{\left(L_{1}^{2}+L_{2}^{2}+L_{3}^{2}\right)}^{2}}\rangle .$$

Here, $L_{1}^{2}$, $L_{2}^{2}$ and $L_{3}^{2}$ are the three eigenvalues of the gyration radius tensor, and 〈⋯〉 denote the ensemble averages. When 〈δ〉=0, the polymer chain has a circular shape; when 〈δ〉 = 1, the polymer chain has a rod-like shape; when 〈δ〉 is between 0 and 1, the polymer chain form ellipses with different long and short half axes.

We plotted the shape factors for type-I and type-II lipid chains in the membranes with $N_{H}1=3$, $N_{T}1=9$, $N_{H}2=3$ and $N_{T}2=9$, as shown in Figure 8c. The shape factors indicated that type-I and type-II lipid chains have almost the same shapes because the two types of lipid chains can be regarded as the symmetric chains with the same polymer parameters. The shape factor slowly increases in the initial stage and then converge into the values nearly 〈δ〉 = 0.89 in the stable stage. Actually, the lipid chains remained unchanged in the adjustment stage. These observations indicated that the two types of lipid chains were rod-like, which are insensitive to the dynamics processes.

For the perforated membranes, we took the same example with $N_{H}1=3$, $N_{T}1=5$, $N_{H}2=3$, and $N_{T}2=6$, as shown in Figure 9. Three dynamics stages were also labelled in the evolutions of gyration radius and shape factors. In these dynamics processes, $\langle R_{gxx}\rangle$ and $\langle R_{gyy}\rangle$ have similar values and exhibit similar behaviour, but their values are greater than those in $\langle R_{gzz}\rangle$. Hence, the lipid chains are compressed along the normal directions of membranes while relatively relaxed along the membrane planes. In particular, the values of $\langle R_{gxx}\rangle$ are greater than those in $\langle R_{gyy}\rangle$ in the initial stage for the type-I lipid chain, but this is an inverse case in type-II lipid chains (Figure 9a,b). In the adjustment and stable stages, $\langle R_{gxx}\rangle$ and $\langle R_{gyy}\rangle$ fluctuate in certain extents and finally converge to similar values; however, $\langle R_{gzz}\rangle$ varies smoothly, reflecting that the chain conformation adjustment occurs along the membrane planes. In contrast to the cases in membranes, $\langle R_{gzz}\rangle$ is smaller than $\langle R_{gxx}\rangle$ and $\langle R_{gyy}\rangle$, indicating that the lipid chains are compressed along the normal directions in the mixed perforated membranes. For the perforated membrane, the shape factor converges into the values nearly 〈δ〉=0.90 in the stable stage (Figure 9c), indicating that type-I and type-II lipid chains are rod-like and insensitive to the dynamics processes, similar to those observed in the membrane case.

### 3.3. Mechanical Properties

We concentrated on the mechanical properties of bilayer membranes and perforated bilayer membranes by analysis of interface tension in this subsection. In the experiments, membrane tension can be measured by various methods [26,28,29]; here, we quantitatively investigated the interface tension of membranes and perforated membranes in the spatial and time domains. According to the Irving–Kirkwood model, the tension along the *z*-direction $\sigma_{z}$ can be expressed as [91]:

(12) $$\sigma_{z}=\langle p_{zz}\rangle -\frac{1}{2}\left(\langle p_{xx}\rangle +\langle p_{yy}\rangle \right),$$

where 〈⋯〉 denotes the ensemble average, and the tensor element $p_{xx}$ is determined by:

(13) $$p_{xx}=\frac{1}{V}\langle \sum_{i=1}^{N_{p}}m_{i}v_{ix}v_{ix}+\sum_{i=1}^{N_{p}}\sum_{ji}^{N_{p}}F_{ijx}x_{ij}\rangle .$$

Here, *V* is the system volume, $N_{b}$ is the number of DPD beads and $x_{ij}$ is the displacement between the *i*-th and *j*-th beads in the *x*-direction. $F_{ijx}$ is the force between the *i*-th and *j*-th beads in the *x*-direction. The elements $p_{yy}$ and $p_{zz}$ are similar to $p_{xx}$, which have the same expressions in Equation (12), only replacing by the corresponding subscripts.

Two examples were shown in Figure 10, with the same parameters of $N_{H}1=3$, $N_{T}1=9$, $N_{H}2=3$ and $N_{T}2=9$ for the membranes and of $N_{H}1=3$, $N_{T}1=5$, $N_{H}2=3$ and $N_{T}2=6$ for the perforated membrane. Interfacial tension $\langle \sigma_{z}\rangle$ was calculated as a function of distance along *z*-direction, as shown in the inserted morphologies, where $\langle \sigma_{z}\rangle$ takes the spatial average over *x*- and *y*-directions within the membrane domains. Here, we observed the nearly zero interfacial tension within the membrane but the no-zero interfacial tensions between the water and lipid domains (Figure 10a). Actually, $\langle \sigma_{z}\rangle$ can be regarded as the line tension defined in the previous works only by a factor [92,93,94], and the liquid–crystal model predicted that the line tension between two domains was not zero in lipid membranes along the membrane surfaces [95]. Our observations are consistent with the previous results, which reminds us it is meaningful to explore how the tension to the membrane surface. In order to observe how the pore affects the interfacial stress, we plotted the interfacial stress near the pore region, i.e., $7r_{c}\leq y\leq 17r_{c}$, as function of the distance along *z*-direction, by taking average over *x*-direction. Indeed, the interfacial stress clearly indicates that the pore can affect the interfacial stress near the pore region since the interfacial stresses have complex distribution along the *z*-direction instead of the double peaks in the membranes. This can be explained by the high curvatures near the pore edges. We observed the interfacial tensions near the pore edges in the perforated membranes (Figure 10c). Hence, the interfacial tensions are non-zero but smaller near the pore, which are due to the different pressures between the water and lipid molecules. The tension depends on lipid tail length and water composition, where the line tensions are the spatial average values [96]. Here, we reported the interfacial tension distributions near the pore regions.

The above observations on the interfacial tensions are limited to the stable pore states, which are not involved in the metastable and unstable pores [25]. Thus, we further investigated the dynamics processes for the interfacial tensions in the membranes with parameters of $N_{H}1=3$, $N_{T}1=9$, $N_{H}2=3$, and $N_{T}2=9$, and perforated membranes with parameters of $N_{H}1=3$, $N_{T}1=5$, $N_{H}2=3$, and $N_{T}2=6$ (Figure 11). Here, we took averages of the interfacial tensions $\langle \sigma_{z}\rangle$ over the whole membrane domains to observe the evolution processes. For the membrane, the results indicated that $\langle \sigma_{z}\rangle =$−0.990, −0.110, and −0.187 in the initial, adjustment and stable stages. The average interfacial tensions converge to non-zero values because they are distributed in the interface between the water and lipid domains. For the perforated membranes, $\langle \sigma_{z}\rangle$ is equal to −0.019, −0.023 and −0.015 in three subsequent stages of dynamics processes, reflecting that the average interfacial tension reaches to nearly zero values and the perforated membranes are tensionless in the stable stage. The previous simulations observed the obvious interfacial tensions near the pores in the perforated membranes with two symmetric layers [39]. The present simulations suggested that the perforated membranes not only have small interfacial tensions near the pores but also are tensionless in the whole membrane due to the mixed assembly of the two types of lipids in the solutions.

## 4. Summary

In this work, we carried out DPD simulations based on CG model to investigate the self-assembly of lipid mixtures in aqueous solutions. Two types of lipid molecules were modelled as the same single-head chains but different single-tail chains with various chain lengths. Several self-assembly structures were observed, whose phase behaviour, structural formation mechanism and mechanical properties were analysed in detail.

Three typical structures, namely, the membrane, perforated membrane and spherical vesicle, were determined and characterised by the chain density distributions and the order parameters. The results indicated that the two types of lipid molecules can cooperatively self-assemble into the mixed structures, where two types of lipid molecules are mixed in each layer. These structures were stable in the phase diagrams constructed by the tail chain lengths $N_{T}1$ and $N_{T}2$, where the spherical vesicles were distributed in the corner while the membranes and perforated membranes were located near the diagonal lines in phase diagram. This finding reflects that similar lipid chains are easier to mix into layer structures with flat surfaces, but the two types of lipids with distinct tail chain lengths tend to form vesicles with spherical surfaces.

The system energy as functions of time suggested that the dynamics processes can be divided into three evolution stages, i.e., the initial stage, the adjustment stage and stable stage. The mixtures between the type-I and type-II lipid chains were clearly demonstrated in these three stages in the dynamics processes. The dynamics processes showed that the $\langle R_{gxx}\rangle$, $\langle R_{gyy}\rangle$ and $\langle R_{gzz}\rangle$ exhibit different types of behaviour for the membranes and perforated membranes in these evolution stages. Specifically, the type-I and type-II lipids have the similar variations of $\langle R_{gxx}\rangle$, $\langle R_{gyy}\rangle$ and $\langle R_{gzz}\rangle$. However, the variations in $\langle R_{gzz}\rangle$ are quite different from those in $\langle R_{gxx}\rangle$ and $\langle R_{gyy}\rangle$ for the membranes and perforated membranes, respectively. The results showed that $\langle R_{gxx}\rangle$ and $\langle R_{gyy}\rangle$ oscillate greatly, but $\langle R_{gzz}\rangle$ varies slowly, indicating that adjustment mostly occur along the surface planes. However, the shape factors $\delta_{1}$ and $\delta_{2}$ are insensitive to the dynamics processes, and the short tail chains easily form rod-like shapes for various types of lipid molecules.

The distribution of interfacial tension along the normal directions of membranes suggested that the presence of non-zero tension near the surfaces between the lipids and water for the membranes and perforated membranes. The interface tension along the membrane planes also suggested such observations where non-zero tension exists near the pores. The interface tensions were also investigated in the dynamics processes. The results indicated that the interface tension near the surface increases with time and then developed into stable values for the membranes and perforated membranes. Our results can be extended to other complex lipid systems and deepen our understanding of the self-assembly mechanism of lipid mixtures in aqueous solutions.

## Figures and Tables

**Table 1:** System parameters in the simulation box.

Box size			$V=L_{x}\cdot L_{y}\cdot L_{z}=30r_{c}\cdot 30r_{c}\cdot 30r_{c}$				
DPD parameters			**σ=3.0** γ=4.5				
$a_{ij}$		Beads	H1 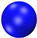	T1 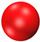	T1 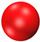	H2 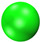	T2 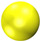
		
Beads							
H1 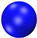			25				
T1 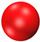			100	25			
W 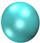			25	100	25		
H2 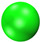			25	100	25	25	
T2 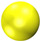			100	100	100	100	25

Here, 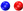 are the head and tail beads of type-I, 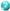 is water molecule, 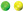 are the head and tail beads of type-II respectively.

## Data Availability

Not applicable.
